# Reconstruction of vertical alveolar ridge deficiencies utilizing a high-density polytetrafluoroethylene membrane /clinical impact of flap dehiscence on treatment outcomes: case series/

**DOI:** 10.1186/s12903-022-02513-7

**Published:** 2022-11-15

**Authors:** Daniel Palkovics, Fanni Bolya-Orosz, Csaba Pinter, Balint Molnar, Peter Windisch

**Affiliations:** 1 Department of Periodontology, Semmelweis University, Szentkirályi street 47 4th floor, Budapest, 1088 Hungary; 2 Empresa de Base Technológica Internacional de Canarias, S.L. (EBATINCA), The Alcalde Jose Ramirez Bethencourt Avenue 17, 35004 Las Palmas De Gran Canaria, Spain

**Keywords:** Wound dehiscence, Membrane exposure, D-PTFE, Vertical ridge augmentation, Guided bone regeneration, CBCT analysis, 3D evaluation

## Abstract

**Objectives:**

The aim of this study was to evaluate the effects of membrane exposure during vertical ridge augmentation (VRA) utilizing guided bone regeneration with a dense polytetrafluoroethylene (d-PTFE) membrane and a tent-pole space maintaining approach by registering radiographic volumetric, linear and morphological changes.

**Methods:**

In 8 cases alveolar ridge defects were accessed utilizing a split-thickness flap design. Following flap elevation VRA was performed with tent-pole space maintaining approach utilizing the combination of a non-reinforced d-PTFE membrane and a composite graft (1:1 ratio of autogenous bone chips and bovine derived xenografts). Three-dimensional radiographic evaluation of hard tissue changes was carried out with the sequence of cone-beam computed tomography (CBCT) image segmentation, spatial registration and 3D subtraction analysis.

**Results:**

Class I or class II membrane exposure was observed in four cases. Average hard tissue gain was found to be 0.70 cm^3^ ± 0.31 cm^3^ and 0.82 cm^3^ ± 0.40 cm^3^ with and without membrane exposure resulting in a 17% difference. Vertical hard tissue gain averaged 4.06 mm ± 0.56 mm and 3.55 mm ± 0.43 mm in case of submerged and open healing, respectively. Difference in this regard was 14% between the two groups. Horizontal ridge width at 9-month follow-up was 5.89 mm ± 0.51 mm and 5.61 mm ± 1.21 mm with and without a membrane exposure respectively, resulting in a 5% difference.

**Conclusions:**

With the help of the currently reported 3D radiographic evaluation method, it can be concluded that exposure of the new-generation d-PTFE membrane had less negative impact on clinical results compared to literature data reporting on expanded polytetrafluoroethylene membranes.

## Background

Advancements in reconstructive periodontal and implant surgery, flap management and material sciences have enabled clinicians to deliver highly esthetic- and functional implant-prosthodontic rehabilitations [1]. Even in severely compromised cases implant retained prosthetic rehabilitation has become a feasible solution. However, vertical ridge augmentation (VRA) can still oppose challenges in certain scenarios due to compromised soft- and hard tissue conditions and to the complexity of flap management.

Distraction osteogenesis, onlay grafting (OG) and guided bone regeneration (GBR) was found to be applicable for VRA [2]. Due to the high complication rate, distraction osteogenesis is only recommended for highly skilled clinicians. OG and staged GBR presented similar results in terms of complication rate (OG: 8,1%, GBR: 6,95%) and implant survival rate (OG: 96,3%, GBR: 100%), with GBR performing slightly better [2]. However, in the case of severely atrophied alveolar ridges intraorally harvested autogenous bone blocks may not present sufficient volumes, therefore, extraoral donor sites could be considered as an alternative with the necessity of hospitalization. The greatest issue following OG is the resorption rate of the graft [3], which in the earlier stages of healing was found to be 24,16% (iliac grafts) and 8,44% (calvarial grafts) [4].

Previously a split thickness flap design for VRA was described by Windisch and co-workers [5, 6]. In their approach vertical- and periosteal releasing incisions were avoided to avoid the disturbance of periosteal blood supply [7]. It was emphasized in previous studies that improved postoperative blood supply may reduce the likelyhood of a membrane exposure [5–7].

For VRA utilizing GBR the application of non-resorbable membranes for space maintenance is widely recommended [8]. Titanium meshes and titanium reinforced polytetrafluoroethylene (PTFE) membranes are used most frequently. These membranes have a semi-ductile structure that can be formed and are able to maintain their shape, thus creating a safe secluded space [6, 9].

Most common complication of GBR is the exposure of the membrane [2]. Negative effects of membrane exposure are generally accepted in the literature, however only a handful of articles compare clinical outcomes of cases with and without complications [10–14]. According to Verardi & Simion [15] complication can be classified into four different categories: (i) class I: small membrane exposure (< 3 mm) with no purulent exudate; (ii) class II: large membrane exposure (> 3 mm) with no purulent exudate; (iii) class III: Membrane exposure with purulent exudate; (iv) class IV: abscess formation without the exposure of the membrane [16].

Previously both high density- and expanded polytetrafluoroethylene (e-PTFE) membranes have been utilized successfully for GBR. The larger pore size of e-PTFE membranes (5–30 μm) might allow for better transmembraneous delivery of nutrients and enhanced graft maturation. Whereas small pore sizes of d-PTFE membranes (0.2 μm) may serve as a more efficient barrier against bacterial penetration and epithelial ingrowth [17]. Despite these contradictory theoretical concepts Ronda and co-workers [18] did not report any significant difference in clinical performance between the two barriers.

Recently a new generation of non-reinforced high-density polytetrafluoroethylene (d-PTFE) membranes have been released (permamem®, botiss biomaterials GmbH, Zossen, Germany), primarily intended for open healing in alveolar ridge preservation procedures [19, 20]. Due to the dense structure, the membrane is impervious to bacteria [21, 22], therefore it can be emphasized that even in case of a membrane exfoliation acceptable outcomes might be achieved. However, without reinforcement to maintain a safe secluded space for regeneration, the membrane support must be provided by means of tenting screws [23, 24].

The aim of this study was to evaluate the effects of membrane exposure during VRA using a new-generation d-PTFE membrane with a tent-pole space maintaining approach by registering radiographic volumetric, linear and morphological changes.

## Materials and methods

### Study design

The present study demonstrates clinical and volumetric radiographic changes of 8 cases following staged VRA utilizing a non-reinforced d-PTFE membrane and supporting tenting screws in conjunction with a split thickness flap design. Selected cases are part of a larger ongoing clinical study. All patients were selected and treated at the Department of Periodontology, Semmelweis University. The study protocol was approved by the Semmelweis University Regional and Institutional Committee of Science and Research Ethics (Approval Number: SE RKEB 145/2018). Surgical interventions were performed with the understanding and written informed consent of every participant. The study was conducted in full accordance with the Helsinki Declaration of 1975, as revised in 2013 [25]. Surgical procedures were performed by two experienced surgeons (BM and PW), segmentation of the CBCT datasets and radiographic measurements were performed by three trained professionals (DP, FBO and CSP).

### Patient selection

**Fig. 1:**
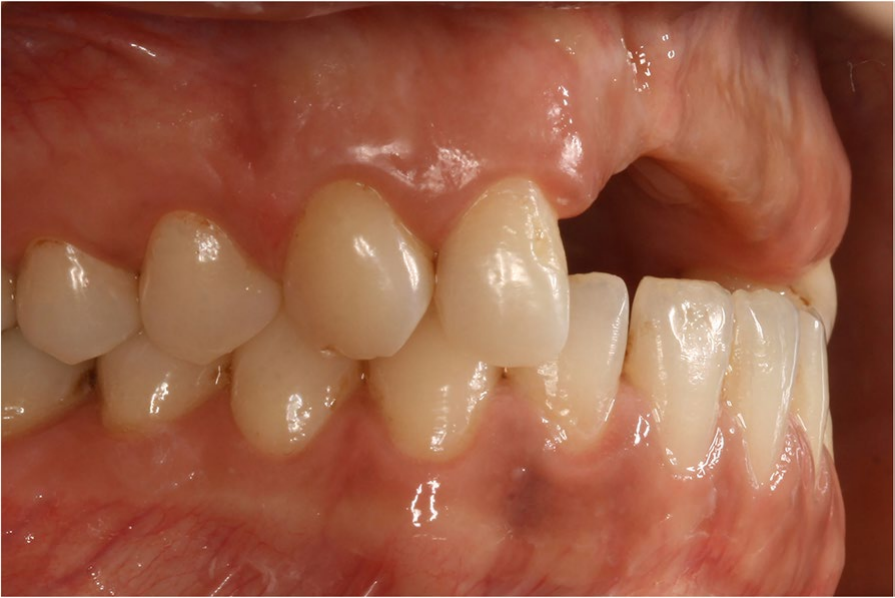
Baseline clinical situation

Patients with good compliance and good oral hygiene were enrolled with at least a single tooth gap where vertical augmentation was necessary to provide proper functional and esthetic outcome for implant retained prosthetics (Fig. 1). Due to the study’s pilot nature severity of vertical ridge deficiencies varied largely between cases. Exclusion criteria were the following: (i) general medical conditions: previous irradiation therapy in the maxillo-facial area, uncontrolled diabetes, systemic steroid treatment, systemic bisphosphonate treatment, pregnant or lactating women, (ii) smoking: only non-smoking patients were enrolled, (iii) periodontal status: untreated periodontitis with high levels of residual inflammation, full mouth bleeding score (FMBS) ≥ 25% [26], (iv) oral hygiene: full mouth plaque score (FMPS) ≥ 25% [27], (iv) patients refusing to sign the informed consent document.

Cone-beam computed tomography (CBCT) images (I-CAT FLX®, KaVo Dental GmbH, Bieberach an de Riß, Germany: 300 μm voxel size; 120 kV anode voltage; 36 mA x-ray tube current) were taken prior and 9 months following VRA procedure [28].

### Surgical procedure

#### Split thickness flap preparation

**Fig. 2:**
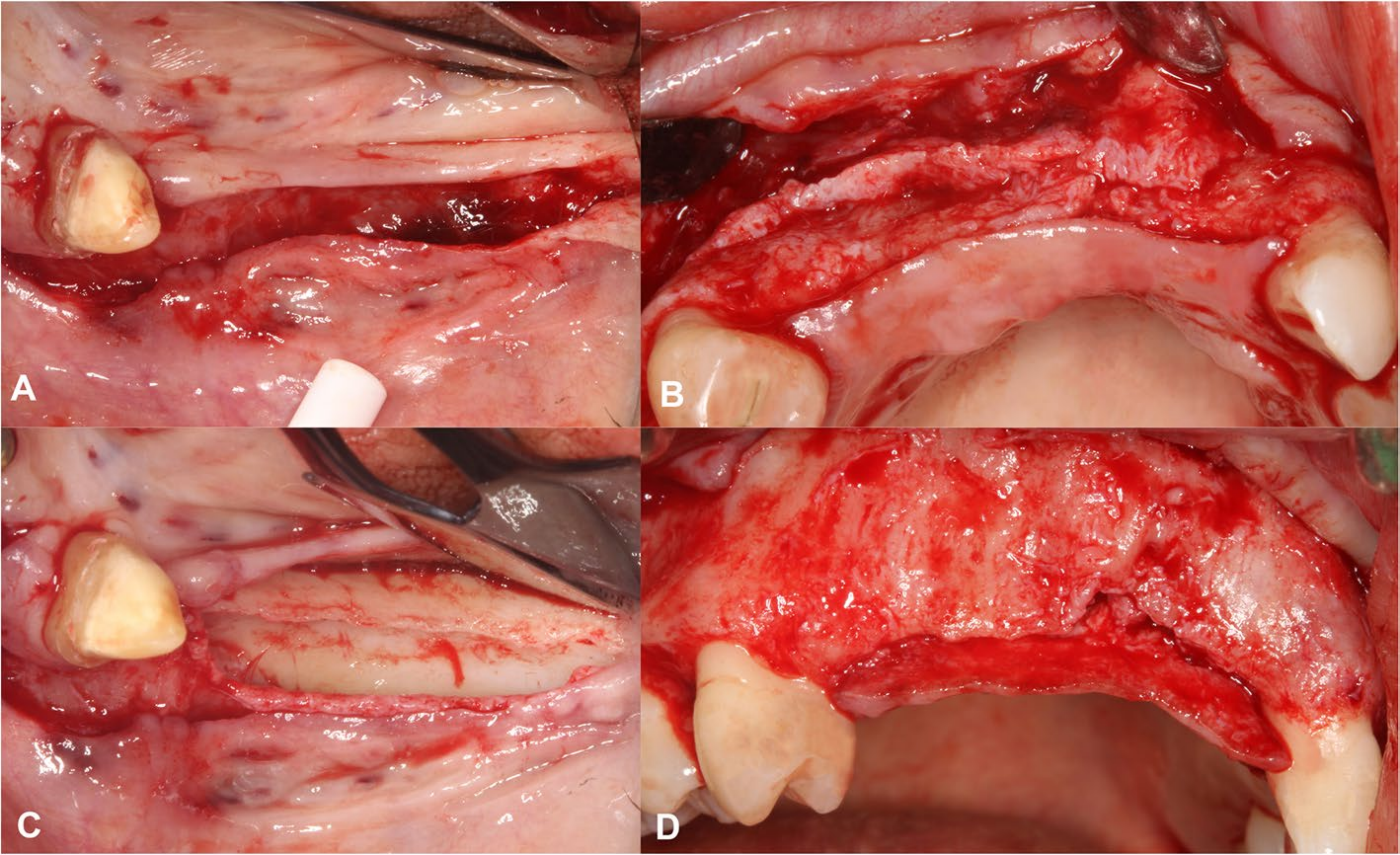
Split-thickness flap elevation. **A** Buccal mucosal layer preparation in a lower lateral region. **B** Buccal mucosal layer preparation in an upper anterior region. **C** Buccal periosteal layer and lingual full thickness flap preparation. **D** Buccal periosteal layer and palatal full thickness flap preparation

A split thickness flap design was utilized as described by Windisch and co-workers [5, 6]. After local anesthesia a midcrestal incision made along the edentulous ridge was extended intrasulcularly at two neighboring teeth for additional flap mobilization. Vertical and periosteal releasing incisions were avoided to avoid the disturbance of periosteal blood supply [7]. On the palatal and lingual aspects, a full-thickness flap was elevated. On the buccal side, a split-thickness flap was carried out as described by Windisch et al. [5, 6]. After the preparation of the mucosal layer the periosteum was elevated from the bone surface by blunt dissection [5, 6] (Fig. 2).

#### Harvesting autogenous bone chips and tenting bone micro blocks

**Fig. 3:**
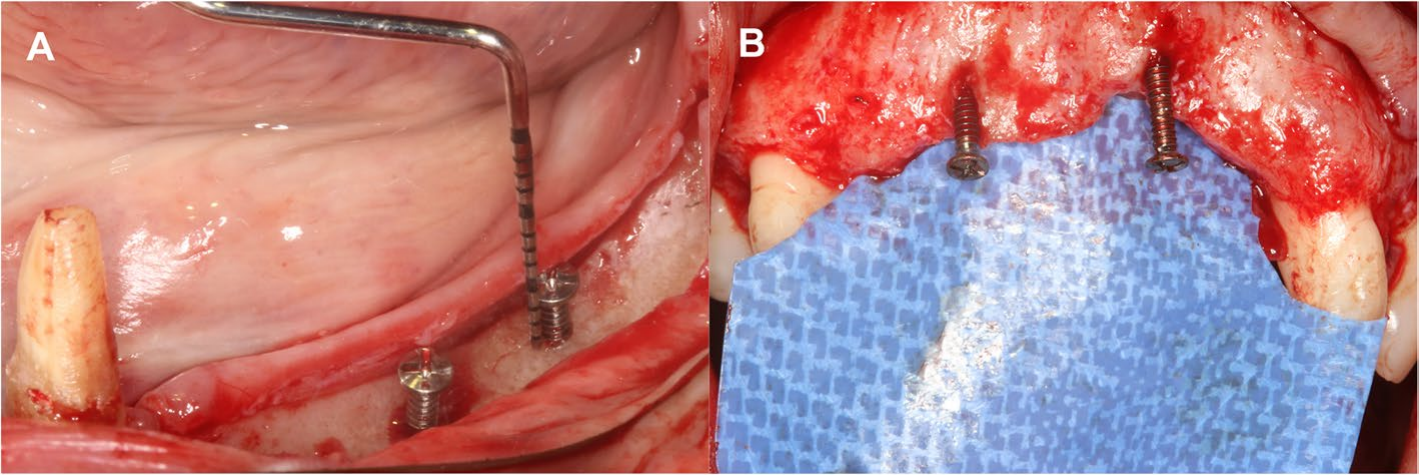
Insertion of tenting screws. **A** Lower lateral region. **B** Upper anterior region

A 50–50% mixture of locally harvested autogenous bone chips and bovine derived xenograft (BDX) particles was applied, thereby a composite graft was obtained [29]. Bone chips were collected by means of a disposable bone collector (SafeScraper Twist®, Meta, Reggio Emilia, Italy) from intraoral donor sites (i.e. mandibular ramus, nasal spine) without the preparation of a second surgical site. Autogenous bone chips were collected in a sterile Petri dish, hydrated with sterile saline solution and mixed with BDX (cerabone®, botiss biomaterials GmbH, Zossen, Germany). Tenting screws (Pro-Fix™ Precision Fixation System, Osteogenics, Lubbock, Texas, USA) were inserted into the edentulous ridge according to the desired vertical bone height (Fig. 3).

#### Membrane adaptation and bone grafting

**Fig. 4:**
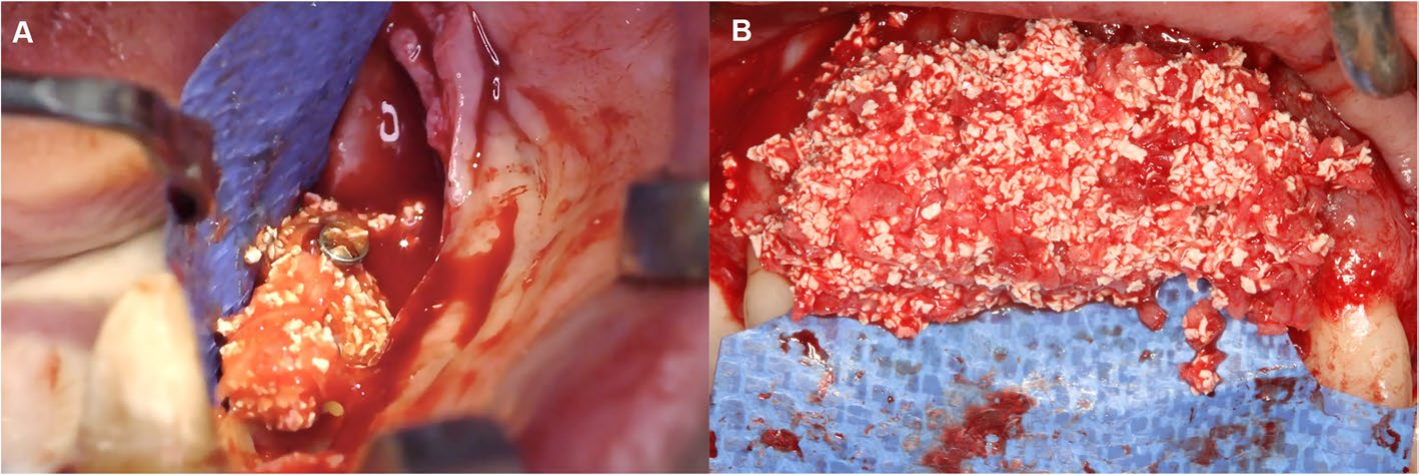
Composite graft (50% autogenous bone chips, 50% bovine derived xenograft) placed around the tenting screws. **A** Lower lateral region. **B** Upper anterior region

**Fig. 5:**
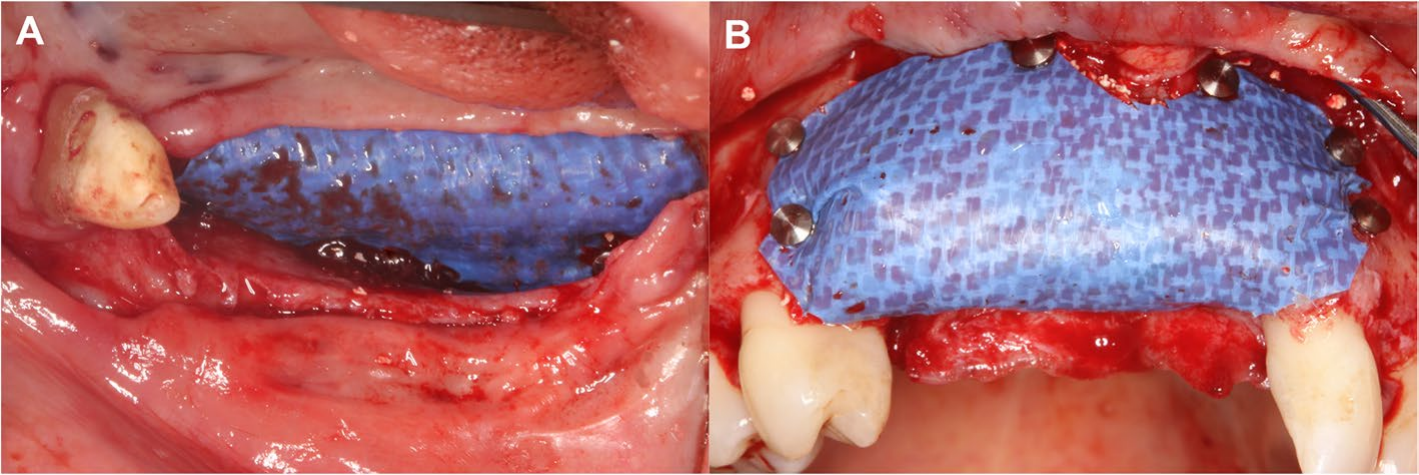
Fixation of the d-PTFE membrane by titanium pins. **A** Lower lateral region. **B** Upper anterior region

The d-PTFE membrane (permamem®, botiss biomaterials GmbH, Zossen, Germany) was shaped according to the size of the vertical ridge defect. First it was fixed on the lingual/ palatal aspect with titanium pins (Titan pin set®, botiss biomaterials GmbH, Zossen, Germany) or membrane fixation screws (Pro-fix Precision Membrane Fixation System®, Osteogenics Biomedical, Lubbock, Texas, USA). After the membrane was secured on the lingual/ palatal side, the composite graft was compacted tightly around the tenting screws (Fig. 4). Following grafting the membrane was folded over the heads of the bone fixation screws and secured on the buccal aspect (Fig. 5).

#### Double layer wound closure

**Fig. 6:**
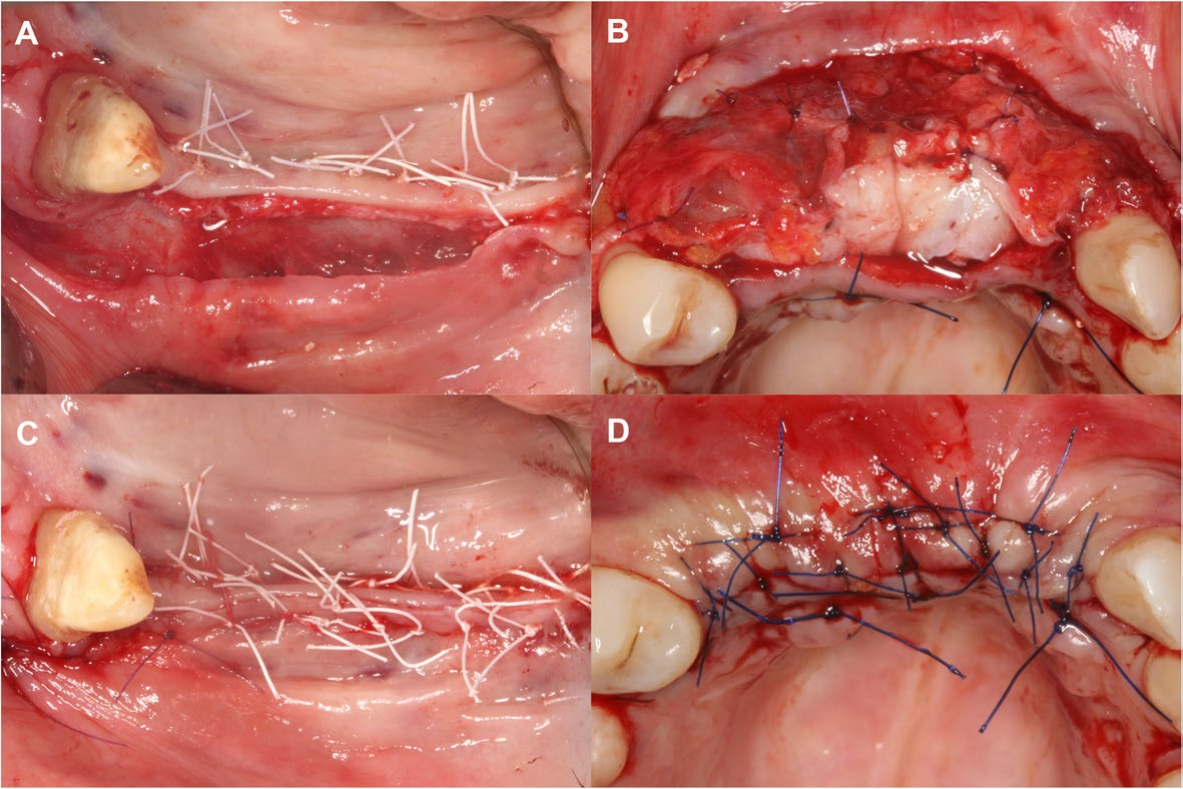
Double layer wound closure. **A** Suturing of the periosteal layer at a lower lateral region. **B** Suturing of the periosteal layer with the addition of a subepithelial connective tissue graft for complete coverage of the membrane at an upper anterior region. **C** Suturing of the mucosal layer at a lower lateral region. **D** Suturing of the mucosal layer at a lower lateral region

Firstly, the buccal periosteum was sutured with horizontal mattress sutures to the lingual/ palatal full thickness flap using a 4–0 non-resorbable monofilament suturing material, knots were tightened on the lingual/ palatal aspect. If complete periosteal coverage of the augmented area could not be achieved, a subepithelial connective tissue graft harvested from the palate and was utilized for coverage (Fig. 6B) [6]. Thereafter, the buccal mucosal layer was sutured to the lingual/ palatal flap with horizontal mattress sutures using a 5–0 non-resorbable monofilament suturing material, knots were tightened on the buccal aspect [5, 6, 30]. Finally, single interrupted sutures were inserted to passively adapt flap margins. Sutures were removed after 14 days (Fig. 6).

#### Postoperative care

Systemic antibiotic treatment was prescribed (500 mg amoxicillin + 125 mg clavulanic acid) for the first postoperative week, three times a day. Patients were advised to avoid brushing at the surgical area for 2 weeks and to rinse with 0,2% chlorhexidine mouth wash (Curasept ADS 220, Curaden International AG, Kriens, Switzerland) two times a day. Patients were recalled 3 days, 1 week and 2 weeks after surgery and regular checkups were held at 1, 3, 6 and 9 months. After a 9-month healing period follow-up CBCT scans were taken, and patients were scheduled for implant placement.

**Fig. 7:**
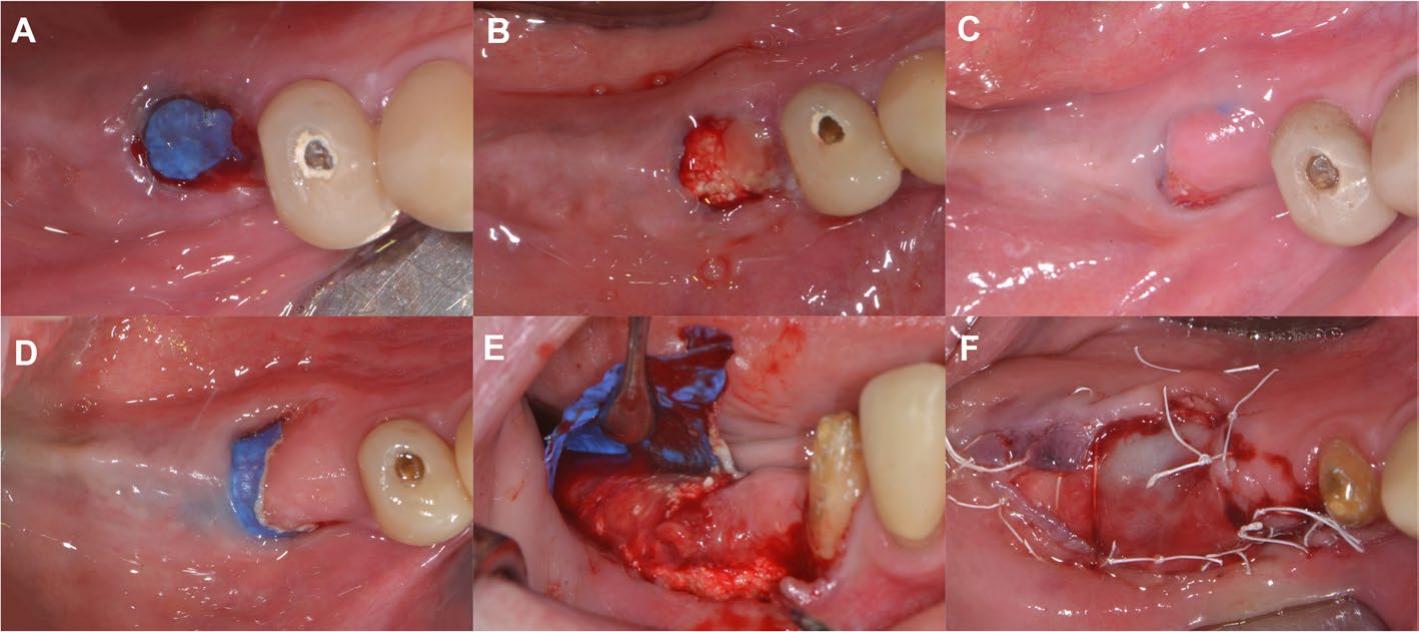
Management of class II membrane exposure. **A** Membrane exposure occurring at the 4th postoperative week. **B** Removal of the exposed area of the d-PTFE membrane. **C** Partial secondary epithelialization at the 6th postoperative week **D** Further progression of membrane exposure at the 8th postoperative week. **E** Flap elevation and membrane removal, stable non inflamed immature hard tissue below the membrane. **F** Coverage of the exposed area using a resorbable collagen membrane

In case of a membrane exposures if no fistula formation and purulent exudates could not be detected the exfoliated portion of the membrane was removed. If secondary epithelialization occurred and covered the exposed area the membrane was maintained for the remainder of the healing period [31]. After removal of the exposed membrane portion, the underlying graft area was irrigated daily with 0.2% chlorhexidine-digluconate. If secondary epithelialization did not cover the denudated area within 3 weeks completely or membrane exposure proceeded the barrier membrane was removed and surgical area was covered with a resorbable collagen membrane (collprotect®, botiss biomaterials GmbH, Zossen, Germany) (Fig. 7).

### Outcome variables

**Fig. 8:**
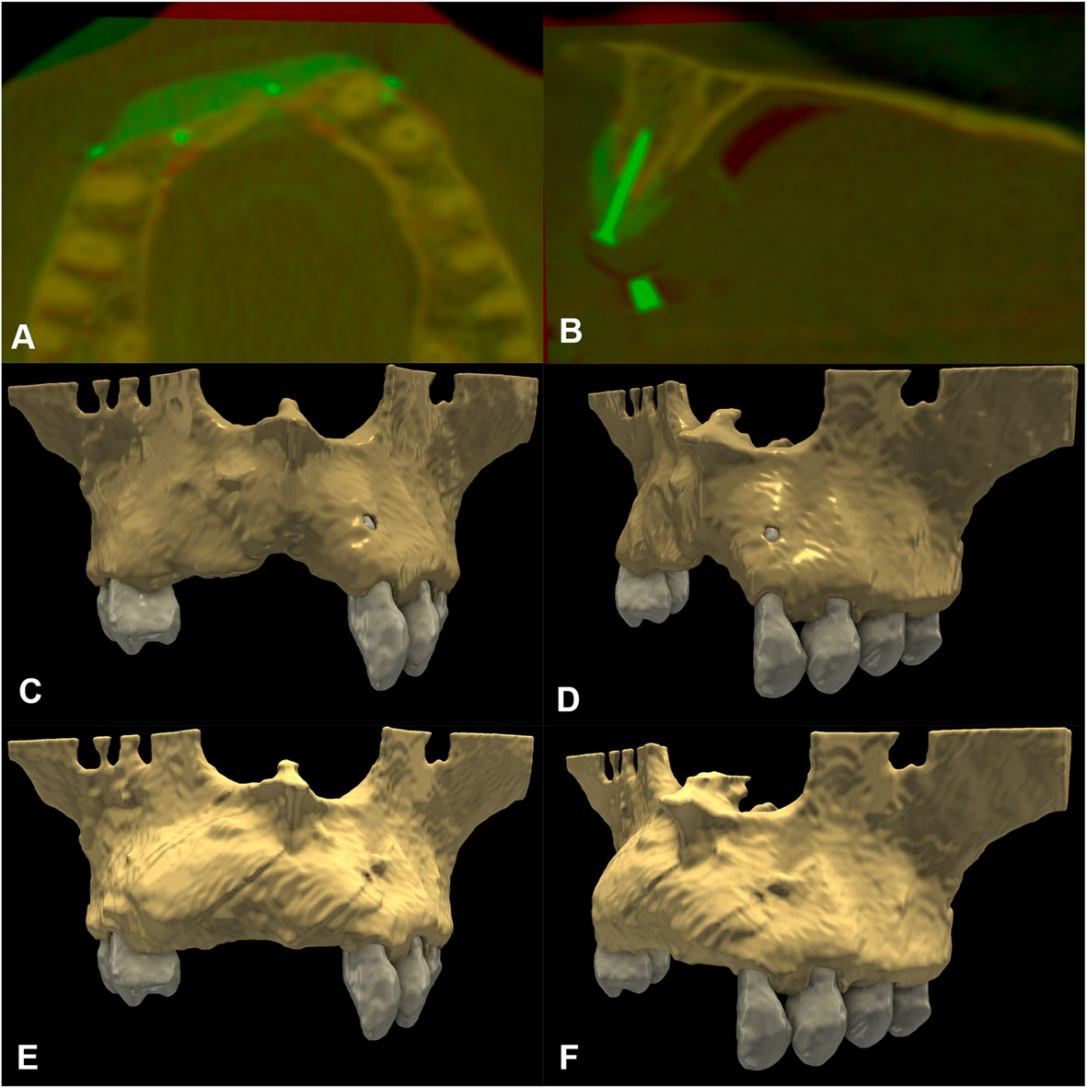
Baseline and 9-month follow-up radiographic situation. **A**-**B** Spatial registration of pre-and postoperative CBCT scans. Color palette has been changed for a better assessment of hard tissue changes (preoperative CBCT: red, postoperative CBCT: green). **C**-**D** Segmented 3D model of the baseline situation. **E**-**F** Segmented 3D model of the 9-moth follow-up situation

**Fig. 9:**
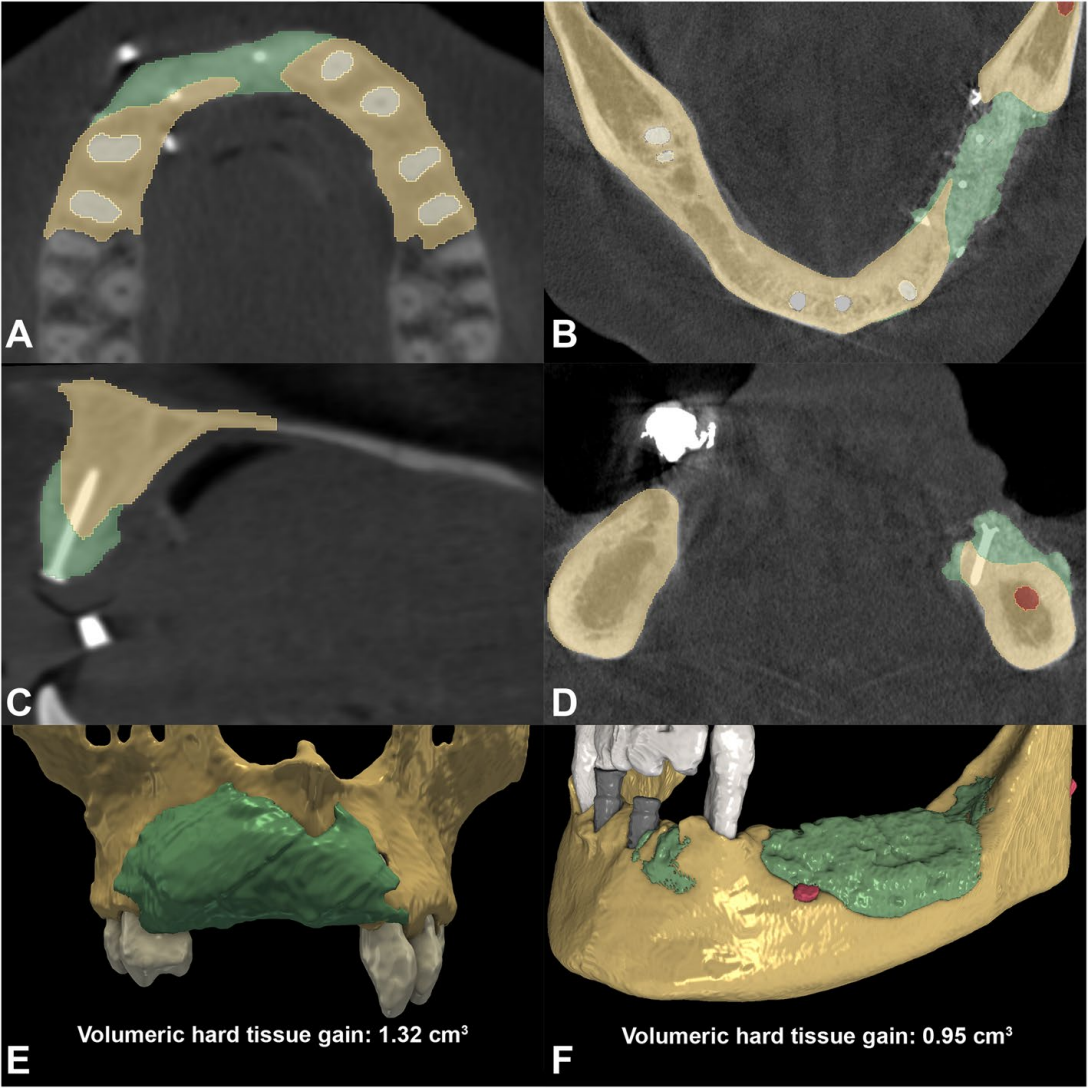
3D subtraction analysis for the quantification of volumetric hard tissue gain. **A**, **C** Planar view of newly formed hard tissues in relation to baseline situation in an upper anterior case (brown label: outline of baseline situation; green label: outline of newly formed hard tissues; white label: outline of teeth). **B**, **D** Planar view of newly formed hard tissues in relation to baseline alveolar ridge dimensions in a lower lateral case (brown label: outline of baseline situation; green label: outline of newly formed hard tissues; white label: outline of teeth; gray label: outline of implants; red label: outline of infra-alveolar canal). **E** 3D view and volumetric calculation of newly formed postoperative hard tissue tissues (Participant 7). **F** 3D view and volumetric calculation of newly formed postoperative hard tissue tissues (Participant 1)

Primary outcome of the study was to assess the volumetric and linear changes following VRA procedures. Evaluation was performed utilizing a semi-automatic image segmentation method and 3D radiographic subtraction analysis as described elsewhere in detail [32, 33]. Briefly, CBCT data were imported into an open-source Digital Imaging and Communications in Medicine (DICOM) imaging software platform (3D Slicer) [34]. With the help of a semi-automatic image segmentation method [32, 33] surgical areas on the pre- and postoperative CBCT scans (edentulous ridge and adjacent teeth) were segmented, subsequently generating 3D virtual models. Segmentation was carried out with the sequence of: (i) delineation of anatomical structures performed on every 10th slice; (ii) morphological contour interpolation to calculate missing labels on in-between slices (iii) smoothing with a kernel size of 5x5x5 mm. Following 3D model acquisition spatial registration of DICOM datasets was performed using an intensity-based medical image registration algorithm [35] (Fig. 8). Once spatial registration and segmentation on both CBCT scans was completed, subtraction of the pre-and postoperative models was done by applying logical operations, allowing to visualize the morphology of newly formed hard tissues [36] (Fig. 9). Volumetric difference between pre- and postoperative CBCT scans was calculated in the *Segment statistics* module of 3D Slicer.

**Fig. 10:**
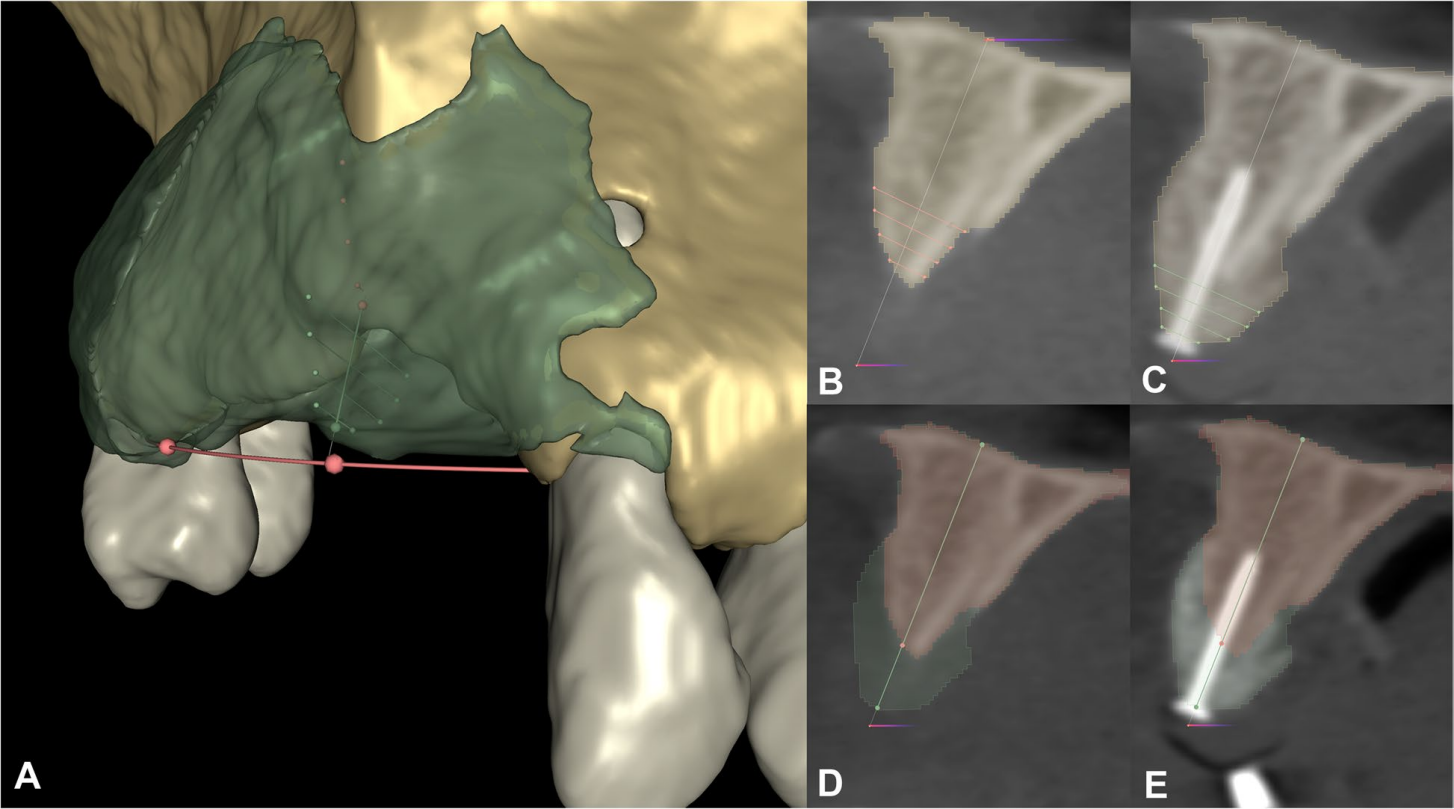
Vertical- and horizontal linear measurements at the greatest of hard tissue gain (Participant 7). **A** Linear measurements visible on a virtual 3D model. Red open curve placed at the level of the proximal bone of adjacent teeth marks the middle point of the alveolar crest. Preoperative measurements are visible in red, postoperative measurements are visible in green. **B**-**C** Baseline and 9-month follow-up horizontal measurements visible on the reoriented planar CBCT image. Horizontal measurements were performed 0,1,2,3 mm-s apical to the marginal crest perpendicular to the white reference line. **D**-**E** Baseline and 9-month follow-up horizontal measurements visible on the reoriented planar CBCT image. Vertical measurements were made along the gray reference line. Preoperative alveolar bone marked with a red, postoperative alveolar bone marked with a green labelmap

Vertical and horizontal linear measurements were performed at the deepest point of the edentulous ridge to assess both absolute baseline and 9-month follow-up values as well as linear hard tissue changes. To assess pre-and postoperative hard tissue dimensions an open curve was traced midcrestally along the upper or the lower dental arch respectively. Markup points were placed at fixed reference points, namely at the level of the proximal bone of teeth adjacent to the surgical site. CBCT datasets were reoriented based on the open curve, subsequently the curve marked the midcrestal point of the edentulous alveolar ridge. A vertical line was placed between the midcrestal point marked by the curve and either the base of the mandible (lower jaw) or the base of the nasal cavity (upper jaw). Vertical measurements to assess baseline and follow-up dimensions were done along this reference line utilizing the *Line markup* tool [36]*.* Horizontal linear measurements were performed 0, 1, 2 and 3 mm-s apical to the alveolar crest at the same aspect (Fig. 10) [37].

Secondary outcome measure was to assess the effects of membrane exposure on healing outcomes by comparing volumetric and linear outcomes of cases with and without exposures.

### Statistical analysis

Due to the small sample size only descriptive statistics were utilized. The percentage of the differences for each variable (volume, horizontal change, vertical change) between the two groups was calculated to determine the level of significance between cases with and without membrane exposure.

## Results

### Patient demographics

Eight patients were enrolled in the present investigation. VRA was performed in three cases at the anterior maxilla and in five cases of the posterior mandible. Due to insufficient ridge height simultaneous implant placement was not applicable in any of the cases. Therefore, a staged GBR approach was the treatment of choice. Six out of eight participants were female, and two patients were male. Mean age was 52.38 ± 17.27 years. Demographic- and surgical site related data are summarized in Table 1.

**Table 1:** Patient demographics and site characteristics

Case	Gender (F^a^/M^b^)	Age	Localization	Surgical area size
1	F	59	Post. Mand.^c^	35, 36, 37
2	M	41	Ant. Max.^d^	11, 21
3	F	72	Post. Mand.	35, 36
4	F	72	Post. Mand.	45, 46
5	M	53	Post. Mand.	45, 46, 47
6	F	62	Post. Mand.	44, 45, 46
7	F	35	Ant. Max.	13, 12, 11, 21
8	F	25	Ant. Max.	11, 12

^a^Female

^b^Male

^c^Posterior mandible

^d^Anterior maxilla

### Baseline- and 9-month linear alveolar ridge dimensions

Baseline and follow-up vertical hard tissue dimensions at the measurement site averaged at 16.88 mm ± 3.37 mm and 20.68 mm ± 3.55 mm respectively. Baseline horizontal alveolar ridge dimensions measure at 0, 1, 2, 3 mm-s apical to the alveolar crest averaged at 1.64 mm ± 0.50 mm, 5.00 mm ± 2.05 mm, 6.93 mm ± 2.34 mm and 8.49 mm ± 2.29 mm respectively. Whereas 9-month follow-up values were 2.16 mm ± 0.76 mm, 4.41 mm ± 0.71 mm, 5.75 mm ± 0.88 mm and 7.51 mm ± 1.11 mm at the same measurement levels respectively. Data are summarized in Table 2.

**Table 2:** Baseline and follow-up vertical- and horizontal linear alveolar ridge dimensions

Case	Vertical dimensions (mm)		Horizontal dimensions measure at different levels apical to the alveolar crest (mm)							
	Baseline	Follow-up	0 mm		1 mm		2 mm		3 mm	
			Baseline	Follow-up	Baseline	Follow-up	Baseline	Follow-up	Baseline	Follow-up
1	15.83	19.90	1.70	2.76	4.58	5.17	6.91	5.25	9.88	8.72
2	12.41	15.58	0.63	0.98	3.42	4.02	4.20	4.36	5.30	5.85
3	19.09	23.21	1.63	3.34	6.63	4.92	9.35	6.47	10.19	7.36
4	15.43	18.65	2.03	2.56	3.63	4.05	5.56	4.79	8.79	7.34
5	23.50	27.20	1.82	1.74	9.44	4.96	10.84	6.82	11.46	9.46
6	18.43	22.52	1.53	1.49	4.22	2.98	8.24	6.47	9.94	7.34
7	15.00	19.71	2.35	2.44	3.83	4.53	5.09	6.10	6.04	6.97
8	15.32	18.66	1.45	1.95	4.23	4.65	5.24	5.82	6.34	7.02
Average ± St. dev.	16.88 ± 3.39	20.69 ± 3.55	1.64 ± 0.50	2.16 ± 0.76	5.00 ± 2.05	4.41 ± 0.71	6.93 ± 2.34	5.76 ± 0.88	8.49 ± 2.29	7.51 ± 1.11

### Radiographic changes

Volumetric and morphological hard tissue alterations were analyzed following spatial registration and 3D subtraction of baseline and 9-months 3D models. Volume of newly formed hard tissues following VRA averaged at 0.76 cm^3^ ± 0.34 cm^3^. Greatest extent of vertical linear hard tissue gain measured midcrestally between baseline and the 9-month follow-up models averaged 3.80 mm ± 0.54 mm. Follow-up horizontal ridge dimensions measured at the same aspect averaged at a 5.75 mm ± 0.88 mm. Data are shown in Table 3.

**Table 3:** Volumetric and linear hard tissue alterations

Case	Volumetric gain (cm^**3**^)	Linear vertical gain (mm)	Linear horizontal gain (mm)	Membrane exposure
1	0.95	4.07	5.25	NO
2	0.51	3.17	4.36	***YES (class II)***
3	0.56	4.12	6.47	NO
4	0.35	3.22	4.79	***YES (class II)***
5	0.93	3.70	6.82	***YES (class I)***
6	0.99	4.09	6.47	***YES (class II)***
7	1.32	4.71	6.01	NO
8	0.45	3.34	5.82	NO
**Average ± St. dev.**	**0.76 ± 0.33**	**3.80 ± 0.54**	**5.75 ± 0.87**	

### Effects of membrane exposure

From the currently reported eight cases three cases presented a class II and one case a class I membrane exposure. The first two postoperative weeks after VRA were uneventful in all cases. None of the patients experienced extreme pain or swelling. Exposures of the d-PTFE membranes occurred between the fourth and sixth postoperative weeks.

In the case of class I exposure the exposed portion of the membrane was excised, secondary epithelialization covered the entire area after 1 week. Further exfoliation of the membrane in this case did not occur, therefore it was maintained until re-entry surgery at implant placement.

In cases of class II membrane exposures, secondary epithelialization of the exposed graft area was incomplete, subsequently exposure of the membrane proceeded. In these cases, membranes were removed between the 6th and 12th postoperative week. Average hard tissue gain was found to be 0.70 cm^3^ ± 0.31 cm^3^ in cases with open healing (membrane exposure) and 0.82 cm^3^ ± 0.40 cm^3^ in cases with submerged healing. In terms of volumetric hard tissue gain a 17% difference could be observed.

Linear vertical hard tissue gain averaged 4.06 mm ± 0.56 mm and 3.55 mm ± 0.43 mm in case of submerged and open healing, respectively, resulting in a 14% difference between the two groups.

Horizontal ridge width at 9-month follow-up was 5.89 mm ± 0.51 mm and 5.61 mm ± 1.21 mm with and without a membrane exposure respectively, resulting in a 5% difference After 9-month follow-up dental implants could be placed at the second stage surgery, additional hard tissue augmentation was not necessary in any of the enrolled cases. Data are summarized in Table 4.

**Table 4:** Effects of membrane exposure on hard tissue alterations

	Without membrane exposure	With membrane exposure	% difference
**Volumetric gain (cm**^**3**^**)**	0.82 ± 0.40	0.70 ± 0.31	**17%**
**Linear vertical gain (mm)**	4.06 ± 0.56	3.55 ± 0.43	**14%**
**Linear horizontal gain (mm)**	5.89 ± 0.51	5.61 ± 1.21	**5%**

## Discussion

In this current investigation vertical ridge augmentation was carried out utilizing a new-generation d-PTFE membrane with a tent-pole space maintaining approach. Three-dimensional hard tissue alterations were analyzed utilizing a semi-automatic image segmentation method and 3D radiographic subtraction analysis [32, 33, 36]. Clinical outcomes and the effects of an eventual membrane exposure were evaluated by comparing radiographic results of open- and submerged healing.

Previously published systematic reviews on VRA reported less favorable outcomes in terms of hard tissue gain in case of membrane exposure. The systematic review by Machtei reported that membrane exposure resulted in 81% less hard tissue gain compared to non-exposed cases [13]. Garcia and co-workers found a statistically significant difference of 31% in vertical bone gain between cases with and without membrane exposure [10]. Cucchi and co-workers found an exposure rate in case of simultaneous VRA and implant placement of 15%, where in case of a complication the membrane, the graft material and all implants had to be removed [11]. In the current case report, class I membrane exposure was treated according to the treatment guidelines described by Fontana and co-workers [16]. However, since complete secondary epithelialization occurred the application of soft tissue augmentation was not necessary. Contrary to the previously suggested guidelines referring to the application of e-PTFE membranes, in the current study, in case of class II membrane exposures after removal of the exposed portion the d-PTFE membrane was maintained in place for at least 6 weeks while patients were recalled regularly for irrigation [31]. Membrane removal of these cases was carried out between the 6th and the 12th postoperative week. In comparison, data from our study have shown that the event of a membrane exposure seemed to have less negative impact on surgical outcomes, possibly related to different surface characteristics of the applied d-PTFE membrane.

The greatest limitation of the current investigation was the small sample size, therefore further comparative evaluation is necessary to investigate the effects and calculate statistical differences of membrane exposure in case of VRA on a larger population.

Due to the small sample no advanced statistical evaluation could be performed, therefore eminent conclusions cannot be derived. However, preliminary data from the current evaluation indicate that with the application of the new generation d-PTFE membrane open healing has a lesser negative effect on surgical results as it was previously reported in the literature for e-PTFE membranes [10, 11, 13].

## Conclusions

With the help of the currently reported 3D radiographic evaluation method, it can be concluded that exposure of the new-generation d-PTFE membrane had less negative impact on clinical results compared to literature data reporting on e-PTFE membranes. It can be emphasized, that the dense structure and small pore size of the new-generation d-PTFE membrane may have contributed to the positive outcomes. However, to acquire more information regarding exposure rate and consequential complications it is necessary to conduct comparative prospective investigations in the future.

## Data Availability

All data generated or analyzed during this study are included in this published article.

## Abbreviations

VRA: Vertical ridge augmentation
OG: Onlay grafting
GBR: Guided bone regeneration
PTFE: Polytetrafluoroethylene
e-PTFE: Expanded polytetrafluoroethylene
d-PTFE: Dense polytetrafluoroethylene
FMBS: Full mouth bleeding score
FMPS: Full mouth plaque score
CBCT: Cone beam computed tomography
BDX: Bovine derived xenograft
DICOM: Digital Imaging and Communications in Medicine
